# Positional contrasts in key techniques between men's and women's basketball at the Olympic level

**DOI:** 10.3389/fspor.2025.1657992

**Published:** 2025-10-21

**Authors:** Yihong Zhang, Jie Wang, Shi Tan

**Affiliations:** School of Athletic Performance, Shanghai University of Sport, Shanghai, China

**Keywords:** basketball techniques, player positions, gender differences, ensemble learning, match analysis

## Abstract

This study aims to indentify the key techniques of role players at each position in the Olympic basketball games, contrast the differences in key technique performance between men’s and women’s basketball players at the different positions, and explore the factors that lead to the differences in technique performance. Comprehensive offensive and defensive technique data were systematically recorded across 52 games during the 2024 Olympic Games. CatBoost algorithm was subsequently implemented to identify key techniques through feature importance analysis. Results showed that the key techniques for men guards were 2PM, 3PM, AST, One-hand Pass, and BLK. The key techniques for women guards were Two-hand Pass, AST, 2PM, 3PM, and ORB. The key techniques for men forwards were Two-hand Pass, 2PM, FTM, 3PM and One-hand Pass. The key techniques for women forwards were 2PM, DRB, Dribble penetration, Two-hand Pass, and One-hand Pass. The key techniques for men’s centers were defensive rebounds DRB, 2PM, One-Hand Pass, AST, and 3PM. The key techniques for women centers were Two-hand Pass, One-hand Pass, FTM, BLK, and DRB. This study suggests that basketball coaches should design different training methods and competition strategies for players in different positions.

## 1 Introduction

The Olympic Games are the world only truly global and comprehensive games. The number of spectators for the basketball games at the Paris 2024 Olympic Games reached 1,078,319, which set a record. Basketball has entered “the small-ball era,” where players’ positional roles are gradually blurred, and the best players often have flexible and diverse offensive and defensive techniques. However, certain teams instruct their players to train and play according to different positional roles, which results in a decline in their competitiveness and a fall in rankings. In the era of big data, ensemble learning methods can be used to identify the key techniques of players at each position on elite teams. This identification is important for guiding players’ technique training and improving the team technique performance.

Differences in positional roles affect the athletic ability of players (1). Research has found that (2) the physical activity demands, and physical fitness requirements different for basketball position. Professional guards, forwards, and centers exhibit distinct variations in their physical performance capabilities. Gasperi et al. (3) collected technique statistics of players and mentioned that center and guard players have different divisions of labor in offense and defense. This finding suggests that the detailed division of labor for players according to the traditional different positions leads to difficulties in learning diverse techniques styles. Jeličić M et al. (4) collected game data for guards, forwards, and centers to examine performance differences among role players at these three positions. Guards demonstrated higher assist rates, while forwards showed performance levels between guards and centers without any advantage. Centers held advantages in blocks and two-point field goals. Another study (5) conducted a differential analysis of performance indicators for players in various positions during the 2017–18 ACB season. The results revealed significant differences in players’ performance across key indicators. Notably, interior players on winning teams excelled in scoring, creating assists, and securing rebounds. In this study (6), significant strengths and weaknesses are observed in the technique performance of players in each position. The traditional concept by player position can no longer meet the development needs of the modern basketball game. In addition, a significant difference exists between the technique performance of men and women players (7). No significant difference is detected between men and women soccer players in the first half time, but men players have a significantly better passing rate than women players in the second half time (8). In terms of scoring efficiency, men outperform women and men players master a greater variety of shots (9). Another study (10) examined the impact of gender differences in shooting performance on game scoring, revealing that men players attempt more two-point shots and demonstrate superior scoring efficiency compared to women players. Researchers collected and evaluated anthropometric measurements from 239 men and 230 women basketball players in Tunisia. The study found that (11) men players outperformed women players in physical fitness and other aspects, with these differences becoming more pronounced with increasing age. In women basketball games (12), three-point shots play a minimal role in determining game outcomes. Mid-range shots are the key factor for women players to win games, but they are not a key indicator influencing men basketball games. The above research indicates that positional differences and gender differences influence players technical performance and game outcomes. Therefore, quantitatively studying men and women players at the different positions in terms of their technique performance is important. Notably, machine learning methods are better than traditional statistical methods in sports analysis (13). CatBoost is an ensemble learning algorithm involving prediction and feature extraction, and it has been successfully applied in several fields such as clinical medicine and computer science (14–16). Contrasted with other mainstream machine learning methods (17), the CatBoost model is optimal in identifying key players and important features of NBA games, and its accuracy is 92%. Meanwhile, the Catboost model can downsize the relevant indicators into a few key indicators, which in turn simplifies the complexity of the model (18). The study (19) employed k-NN, LB, SVM, Random Forest, and CatBoost to predict outcomes of Greek league soccer matches. Results indicate that the CatBoost model achieved the highest accuracy at 67.73%. To forecast stadium attendance (20), researchers constructed linear regression, CART, Random Forest, CatBoost, and XGBoost models. By evaluating each accuracy and effectiveness of model across five indicators, CatBoost demonstrated high efficacy in predicting game attendance. In the prediction task, the CatBoost model predicts not only the number of goals scored by a team but also the probability of winning, drawing, and losing (21). Therefore, the CatBoost model has strong capability in predicting game results and extracting features.

In summary, players’ gender and positional differences affect their technique performance in the game, and the CatBoost model can accurately identify the team key techniques. However, previous studies still exhibit certain limitations. They chose a single offensive and defensive techniques, which causes difficulty in fully revealing the technique performance that affects the game. Therefore, the study focuses on the basketball tournament at the 2024 Paris Olympics, collects data on the offensive and defensive techniques of men and women basketball players at different positions, and constructs a CatBoost model to achieve the following objectives. The key offensive and defensive techniques affecting the scoring of men and women guard, forward, and center players are identified using the CatBoost model.

1. The key offensive and defensive techniques that affect the game scoring of men guards, forwards, and centers and women guards, forwards, and centers are determined.
2. The differences in key offensive and defensive techniques between men and women basketball players at the different positions are contrasted.
3. The influencing factors that may lead to differences in key offensive and defensive techniques between men and women basketball players in the different positions are explored.

This study aims to provide a method for CatBoost modeling to identify players’ key techniques. It provides a reference for the training of offensive and defensive techniques of men and women basketball players at three positions to help teams increase their game scores.

## 2 Materials and methods

### 2.1 Sample

The study collected data on 13 offensive and defensive techniques from the basketball games at the 2024 Paris Olympics. Table 1 shows the 2024 Paris Olympics basketball tournament comprises three competition phases: the group, knockout, and final, featuring a total of 52 games. Both the men and women tournaments consist of 26 games, with 12 teams competing in each division. The game data were divided into six different datasets based on the different positions of men and women basketball players. The data and their videos were obtained from the official FIBA website and the official website of the Olympic. Offensive Technique Indicators: 2PM is the number of 2-point field goals made by a player in a game. 3PM is the number of 3-point field goals made by a player in a game. FTM is the number of free throws made by a player in a game. ORB is the number of times a player has successfully grabbed a rebound in front of the basket in a game. Two-Hand Pass means a player flicks the ball out with both hands while a teammate receives the pass smoothly. One-Hand Pass indicates a player releases the ball with one hand while a teammate receives the pass in a smooth motion. Dribble penetration means a player uses different dribbling styles to dribble breaks to the basket from the outside to the inside to score or to make at least one free throw after being fouled by opponent shot. Spot-up dribble drive indicates when a player receives the ball, stands still, uses a crossover step or different-side step to start suddenly, and drives from the outside to the inside to score directly breaks to the basket or make at least one free throw Being fouled by opponent shot. Defensive technical Indicators: DRB is the number of times a player successfully grabbed a post rebound during the game. AST is the number of times a player passed the ball to a teammate during the game while the teammate scored directly after the pass was recorded as an assist. STL is the number of times a defensive player hit or slapped the ball from an offensive player hand. BLK is the number of times a defensive player touched the ball by slapping or hitting the ball while an offensive player was shooting and successfully blocked the opponent shot.

**Table 1:** Competition phases and teams for the 2024 Paris Olympic basketball tournament.

Gender	Competition phases	Games
Men	Group	18
	Knockout	7
	Final	1
Women	Group	18
	Knockout	7
	Final	1

Table 1 shows the competition phase (Group, Knockout, and Final) and participating teams for the men and women basketball games at the 2024 Paris Olympics.

### 2.2 Reliability and validity of data

To ensure the reliability of data collection, this study employed retest reliability and inter-observer reliability tests. Specifically, the same observer randomly selected one man and one women game for a second data collection 14 days after the initial collection of a game data. The results of the two data collection sessions underwent a Kappa consistency test. Results showed Cohen kappa values were ≥0.96 for women games, and for men games were ≥0.91, indicating high consistency in data collected by the same observer at different time. Additionally, a national-level basketball player was invited to participate in data collection. First, both observers thoroughly discussed criteria and collection methods before independently gathering relevant data. Cohen kappa coefficient was used to assess the consistency between the data collection of two observers. Results showed that the Cohen kappa value for women games were ≥0.91, and for men games were ≥0.87, indicating high consistency in data collection between the two observers. This provided reliable data support for the research. The study was approved by the Academic Committee of School of Athletic Performance of Shanghai University of Sport.

### 2.3 Methods

#### 2.3.1 Catboost algorithm

**Figure 1:**
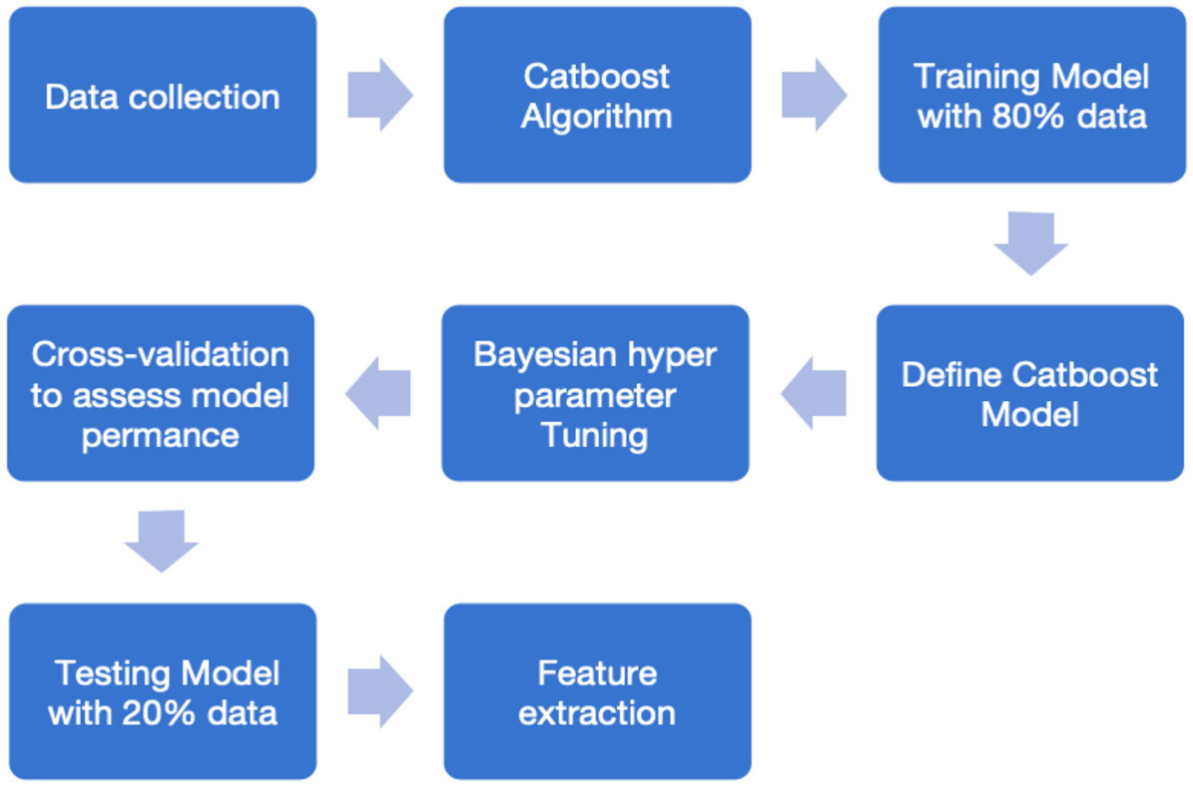
Main steps in modeling. Figure 1 shows the main steps for constructing a Catboost model, including data collection, importing algorithm packages, splitting the dataset, defining the Catboost model, Bayesian hyperparameter tuning, cross-validation, evaluating model performance on the test set, and feature extraction.

CatBoost was an ensemble learning algorithm for Gradient Boosted Decision Trees with high robustness and fast prediction speed. The models were constructed to find the relationship between offensive and defensive techniques and the score of the game; the input layer was the data of offensive and defensive techniques, and the output layer was the score of the game. This process was implemented in Python, and the main steps are shown in Figure 1.

$$F(x)=\sum_{t=1}^{T}\alpha \;t\cdot ft(x)$$

An initial model was established using the target mean. A decision tree was then constructed, the residuals of the current model were calculated, and a new decision tree was built to fit these residuals. This tree was added to the model to reduce the error. Through repeated iterations, decision trees were progressively added until a preset number of trees was reached, or other stopping conditions were met. The initial prediction value was set as the mean value of the score. At each iteration, the difference between the current predicted value and the actual score was calculated. The principle of the symmetric decision tree was applied, selecting the optimal splitting point based on the attack- and defense-technique indicators. The model was updated with the new trees until a preset number of iterations was reached. Bayesian optimization was used to find the optimal hyper-parameter combination. The model was evaluated with cross-validation techniques, and its performance was then tested on a test set. Eventually, the model automatically calculated the importance of the features.

#### 2.3.2 Feature importance

The CatBoost model automatically calculated the feature importance of each attack and defense technique. The sum of the importance values for all indicators was 100. These values were then ranked in descending order; a larger value indicated a greater contribution to the game score, and vice versa. As reported in (22), selecting 40% of all features was considered a reasonable approach, so the five most important indicators were retained as the key attack and defense techniques in the present study.

## 3 Results

This section presents the key offensive and defensive techniques of men and women guards, forwards, and centers and their corresponding feature importance. The results of the contrast between men and women basketball players at the different positions in terms of key techniques are also presented.

### 3.1 Key techniques for men basketball players at different positions

We identified the key offensive and defensive techniques that affect the ability of men guards, forwards, and centers to score game points. Table 2 shows the key techniques for men basketball players at different positions. The results show that 2PM, 3PM, AST, One-Hand Pass, and BLK are the key techniques for men guards. Two-hand Pass, 2PM, FTM, 3PM, and One-hand Pass are the key techniques affecting the scoring of men forwards. In addition, key techniques for men centers are found in offense and defense. These key techniques include DRB, 2PM, One-hand Pass, AST, and 3PM. The key techniques for men forwards are mainly on the offensive side of the ball, while those for men guards and centers are primarily on the offensive and defensive sides of the ball.

**Table 2:** Key techniques for men basketball players at different positions.

Positions	Indicators	Feature importance
Guard	2PM	15.48
	3PM	15.18
	AST	12.87
	One-hand Pass	9.41
	BLK	8.71
Forward	Two-hand pass	38.59
	2PM	10.16
	FTM	8.07
	3PM	7.31
	One-hand pass	6.93
Center	DRB	28.36
	2PM	14.80
	One-hand pass	14.71
	AST	8.87
	3PM	8.30

Table 2 shows that key technique indicators for identified men players in guard, forward, and center positions, along with corresponding feature importance.

### 3.2 Key techniques for women basketball players at different positions

We identified the key offensive and defensive techniques that affect the ability of women guards, forwards, and centers to score in games. Table 3 shows the key techniques for women players at different positions. The results show that Two-hand pass, AST, 2PM, 3PM, and ORB are the key techniques affecting the scoring of women guards. Key techniques for women centers include 2PM, DRB, Dribble penetration, Two-hand Pass, and One-hand Pass. Two-Hand Pass plays an important role in the scoring of women centers. The other key techniques are One-Hand Pass, FTM, BLK, and DRB.

**Table 3:** Key techniques for women basketball players at different positions.

Positions	Indicators	Feature importance
Guard	Two-hand pass	32.66
	AST	23.57
	2PM	6.57
	3PM	6.16
	ORB	5.85
Forward	2PM	23.89
	DRB	16.16
	Dribble penetration	16.11
	Two-hand pass	8.88
	One-hand pass	7.07
Center	Two-hand pass	33.43
	One-hand pass	13.58
	FTM	13.25
	BLK	11.14
	DRB	9.53

Table 3 shows that key technique indicators for identified men players in guard, forward, and center positions, along with corresponding feature importance.

### 3.3 Contrasting the key techniques of men and women basketball players at different positions

We contrasted key offensive and defensive techniques among men and women basketball players across different positions and further visualized the results using radar figures.

**Figure 2:**
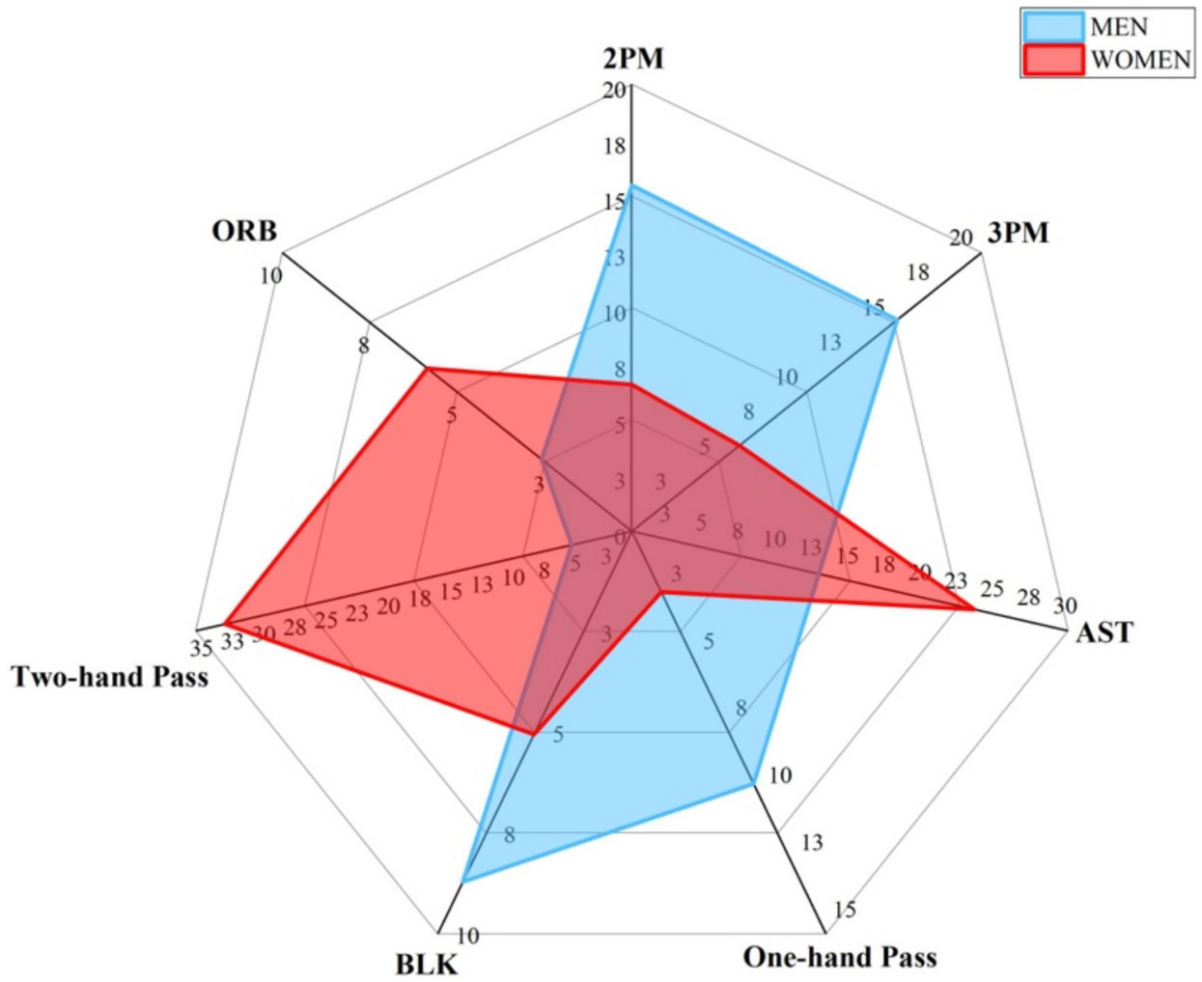
Differences of key techniques for guard position. Figure 2 shows the contrast results of key technique indicators between men and women players in guard position.

Figure 2 shows the comparison of key offensive and defensive techniques between men and women guard players. The 2PM for men guards is 15.48, while the 2PM for women guards is 6.57. Men guards have a 2PM feature importance 8.91 higher than women guards. The 3PM for men guards is 15.18, while the 3PM for women guards is 6.16. Men guards have a 3PM feature importance 9.02 higher than women guards. The AST for men guards was 12.87, while for women guards it was 23.57, indicating men guards had a 10.7 lower AST feature importance than women guards. The BLK for men guards is 8.71, while for women guards it is 5.05. The feature importance of BLK for men guards is 3.66 higher than for women guards. The Two-hand Pass for men guards is 4.86, while for women guards it is 32.66. The feature importance of Two-hand Pass for men guards is 27.80 lower than for women guards. The ORB for men guards is 2.59, while the ORB for women guards is 5.85. The feature importance of ORB for men guards is 3.26 lower than that for women guards. Overall, men and women guard exhibit differences in key techniques aspects at 2PM, 3PM, AST, BLK, Two-hand Pass, and ORB.

**Figure 3:**
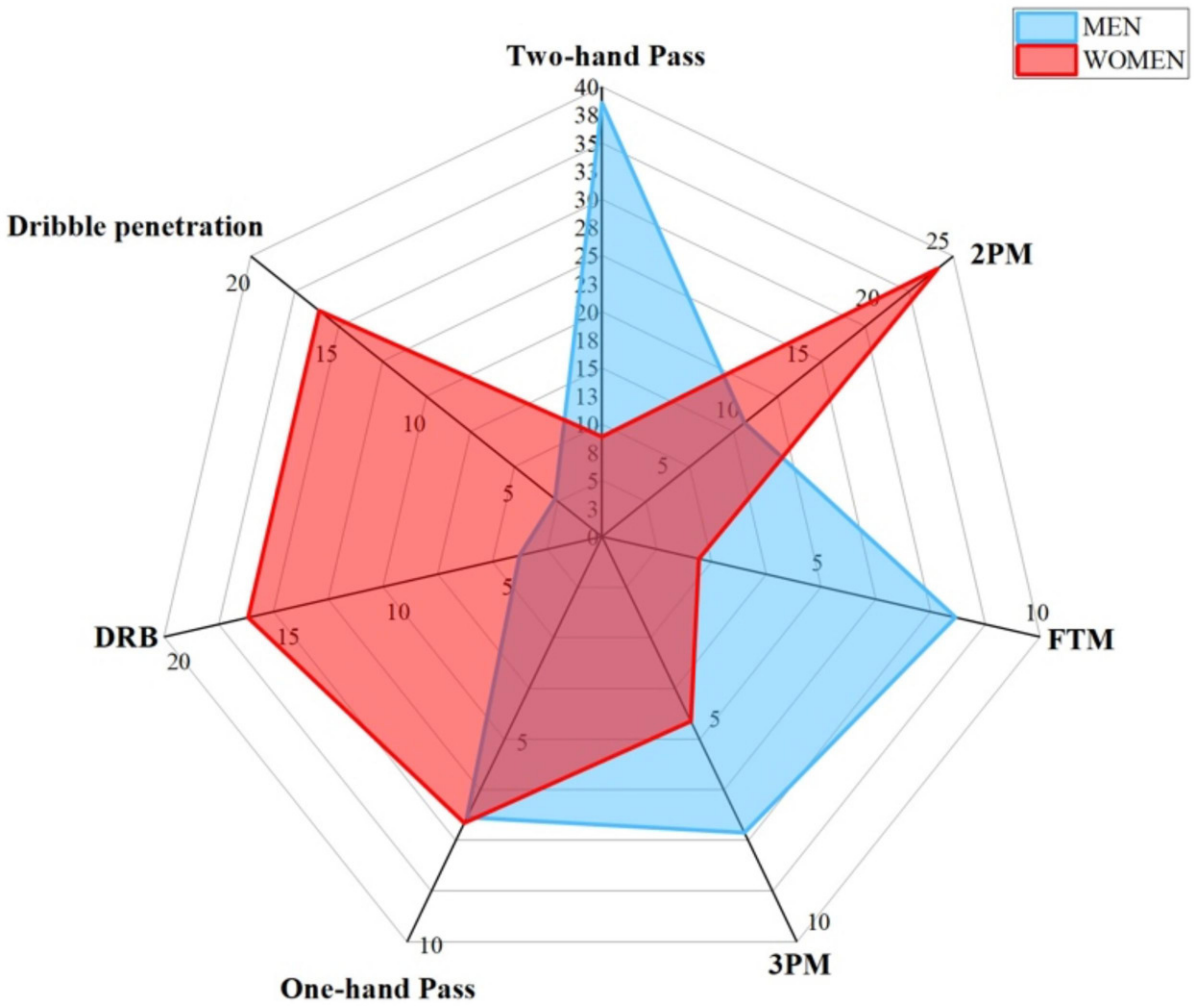
Differences of key techniques for forward position. Figure 3 shows the contrast results of key technique indicators between men and women players in forward position.

Figure 3 shows the results of contrasting the key offensive and defensive techniques of men and women forward players. The Two-hand Pass for men forwards is 38.59, while for women forwards it is 8.88. The Two-hand Pass feature importance is significantly more important for men forwards than women forwards by as much as 29.71. The 2PM for men forwards is 10.16, while for women forwards it is 23.89. The 2PM feature importance is less important for men forwards than female forwards by 13.73. The One-hand Pass for men forwards is 6.93, while for women forwards it is 7.07, indicating men forwards have a 0.14 lower significance for the One-hand Pass feature importance. The FTM for men forwards is 8.07, while for women forwards it is 2.22. The feature importance of FTM for men forwards is 5.85 higher than that for women forwards. The 3PM for men forwards is 7.31, while for women forwards it is 4.56. The feature importance of 3PM for men forwards is 2.75 higher than that for women forwards. The DRB for men forwards is 3.77, while for women forwards it is 16.16. The feature importance of DRB for men forwards is 12.39 lower than that for women forwards. The Dribble penetration for men forwards is 2.68, while for women forwards it is 16.11. The feature importance of Dribble penetration for men forwards is 13.6 lower than that for women forwards. Overall, men and women forward exhibit differences in key techniques aspects at Two-hand Pass, 2PM, One-hand Pass, FTM, 3PM, DRB, and Dribble penetration.

**Figure 4:**
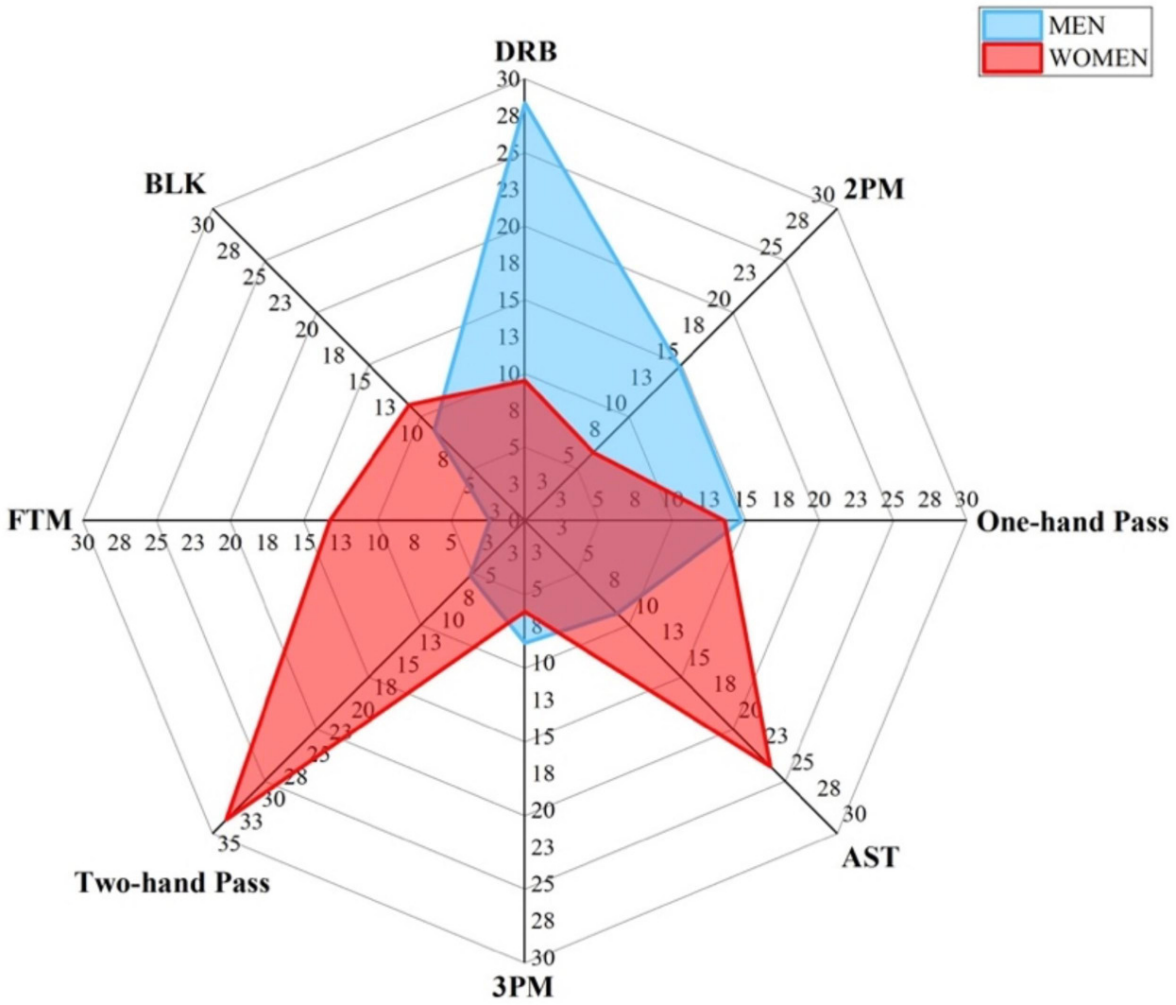
Differences of key techniques for center position. Figure 4 shows the contrast results of key technique indicators between men and women players in center position.

Figure 4 shows the results of contrasting the key offensive and defensive techniques of men and women center players. The DRB for men centers is 28.36, while that for women centers is 13.58. The men center DRB has a feature significance 14.78 higher than the women center. The One-hand Pass for men centers is 14.71, while for women centers it is 13.58, indicating men centers have a 1.13 lower One-hand Pass feature importance than women centers. The 2PM for men centers is 14.80, while the 2PM for women centers is 9.42. The feature importance of 2PM for men centers is 5.38 higher than that for women centers. The AST for men centers is 8.87, while the AST for women centers is 0.00. The feature importance of AST for men centers is 8.87 higher than that for women centers. The 3PM for men centers is 8.30, while the 3PM for women centers is 1.39. The feature importance of 3PM for men centers is 6.91 lower than that for women centers. The FTM for men centers is 2.38, while the FTM for women centers is 13.25. The feature importance of FTM for men centers is 10.87 lower than that for women centers. The BLK for men centers is 4.88, while the BLK for women centers is 11.14. The feature importance of BLK for men centers is 6.26 lower than that for women centers. Overall, men and women center exhibit differences in key techniques aspects of DRB One-hand Pass, 2PM, AST, 3PM, FTM, and BLK.

## 4 Discussion

This study aimed to use the CatBoost model for identifying key offensive and defensive techniques that affect game scoring for men and women basketball players at different positions. The differences in key offensive and defensive techniques between men and women basketball players at the different position were also contrasted. The study found: men and women guards exhibit differences in key techniques aspects at 2PM, 3PM, and AST. Men and women forward exhibit differences in key techniques aspects at Two-hand Pass, 2PM, and One-hand Pass. Men and women centers exhibit differences in key techniques aspects of DRB and One-hand Pass.

The types of shoots of men guards are not only diverse but also exhibit higher shooting percentages (23). In terms of 2PM and 3PM, men guards demonstrate a more pronounced advantage than women guards. This aligns with the perspective of the study (24), which suggests guards positively contribute to game outcomes through higher 2PM and 3PM. This may be linked to home-court advantage, where home-team guards exhibit more aggressive play (25). The study also noted that NBA men guards focus more on ORB, though this finding contradicts our results. Such discrepancies may stem from rule differences affecting player performance (26). The FIBA rules used in the Olympics do not include a defensive three-second rule, whereas NBA rules explicitly enforce it, making it more difficult for interior players to secure offensive rebounds in the paint. Men guards demonstrate superior three-point shooting ability compared to women guards, consistent with the findings of this study (27). The three-point line is 6.75 m from the basket. Women players possess weaker muscular strength and struggle to sustain prolonged physical exertion against fatigue (28), potentially leading to a gradual decline in their three-point shooting accuracy during extended games. Men guards demonstrate superior shot-blocking ability compared to women guards, likely due to differences in physical fitness indicators and certain anthropometric indicators between men and women players (29). However, women guards hold a significant advantage in agility and speed (30), which may explain their greater ability to secure offensive rebounds and increase team scoring compared to their men counterparts. Additionally, women guards outperform men guards in assists. This could be attributed to the higher intensity of Olympic competition, where women guards often assume more orchestrating roles on offense (31).

During the free-throw phase, players may be affected by external distractions, which can impact their free-throw performance (32). This may indicate that men players demonstrate greater psychological resilience when coping with distractions (33). Top level teams often convert defensive rebounds directly into points. Women forwards outperform men forwards in defensive rebounding, exhibiting exceptional agility. Men forwards poorer performance may stem from frequent possession changes during games increasing players’ physiological demands and fatigue (34), thereby reducing efficiency in contesting defensive rebounds. Women forwards possess two-point shooting capabilities and enabling consistent execution of two-point shooting techniques (35). Women forwards contribute more two-point scoring to games than men forwards. This difference may stem from men forwards covering greater distances at high speeds and engaging in multiple off-ball movement tactics (36), where accumulated fatigue impacts two-point conversion rates. Additionally, the slightly superior three-point shooting ability of men forwards compared to women forwards stems from biological differences. Specifically, female forwards struggle to maintain efficient three-point shooting in high-intensity games (37).

Men centers secure more defensive rebounds than for women centers, positively impacting team scoring (38). This may stem from men centers superior lateral jumping and speed capabilities (39). Women centers contribute less to game scoring through defensive rebounding, likely due to cumulative neuromuscular strain from higher per-minute contact and jumping actions (40), leading to reduced jumping frequency and efficiency. Conversely, women centers excel at assists, typically serving as playmakers for teammates while lacking individual scoring options (41). Research on basketball talent identification indicates that (42) creating scoring opportunities and individual offensive capabilities are key indicators for evaluating a player cross-position role. Therefore, men centers should enhance their playmaking abilities and defensive reading skills to further strengthen their assist capabilities. Women centers should further develop and enhance their individual offensive capabilities. This disparity may stem from a significant correlation between game performance and physical demands in men centers (43), leading to cumulative fatigue. women centers contribute more to the game through free throw accuracy than men centers, which contradicts the findings of this study (44). This discrepancy may stem from men centers enduring sustained high-intensity activity during games, where increased internal load likely contributes to lower free throw conversion rates (45, 46). Additionally, women centers demonstrate superior assist capabilities compared to their men counterparts. Assists are decisive in determining game outcomes and represent a key winning indicator for both strong and weak teams at high levels of basketball competition (47). Very few studies have examined the differences in assist capabilities between men and women forwards, yet this is significant in the small-ball era.

In addition, a significant difference exists in the types of passes made by men and women basketball players, which is consistent with the results in (48). Players choose different types of passes when facing defenses of different intensities. One-handed passing can be more appropriate when defending against a pressing defense, and two-handed passing is suitable when defending against a less pressing game. However, no comparisons have been found between men and women basketball players in the different positional roles in terms of two- and one-handed passing. This result may be due to the more cumbersome statistical process of passing technique. Dribble penetration with the ball is an important offensive technique, but fewer studies have been conducted on it. The greater dribbling breakthrough ability of women forwards may be due to that the external load of their in the game is generally smaller than that of men forwards, which implies that they are subjected to relatively small external loads and can distribute their physical energy more efficiently to maintain a good breakthrough efficiency. Meanwhile, men forwards have weaker dribbling control ability, and their taller height leads to higher center of gravity when dribbling. As a result, they have difficulty scoring through direct breakthrough technique. If fouled during while shooting, then they can obtain free throw opportunities. Center players have higher passing stability. In the small-ball era, center players are required to share the offensive task or even serve as the core of the tactics. The use of different passing methods can enable them to effectively coordinate with teammates and achieve the tactical purpose.

The study selected only 13 offensive and defensive technique indicators. This limited number of indicators may overlook players’ performance in certain techniques aspects. Future researchers should categorize fundamental indicators based on types of techniques. For instance, types of shots can be categorized as jump shot, layup, dunk, putback tip-in, alley-oop, and other types. Expanding the dataset by increasing the number of technique indicators will help capture more detailed performances in both offensive and defensive play. Although the algorithm we selected is more advanced, heterogeneity may exist in the results obtained using different algorithms when faced with the different dataset. In addition, the presence of covariates outside of game time (e.g., player sleep, travel distance, stadium conditions, and climate temperature) may have a potential impact on the technique performance of players during the game. Obtaining data on indicators was beyond the scope of this study. Therefore, we did not need to control for their impact on this study.

Future research could focus on basketball technique types to identify additional offensive and defensive indicators. By collecting extensive data on these indicators, the dataset can be expanded to more accurately determine players’ key techniques. At the same time, the effects of other covariates on the technique performance of men and women in different positional roles were considered. On this basis, the reliability of the data and the accuracy of the findings will be further improved.

## 5 Conclusion

This study employed the CatBoost model to identify key offensive and defensive techniques for men and women basketball players across different positions. This study contrasted technique performance across differences positions and genders, examining the factors contributing to these differences. The results showed that differences existed in key technique performance between men and women guards, forwards, and centers. This study provides a feasible research method for the field of basketball game analysis. It also provides a reference for the technique training of players with different positional roles and the enhancement of the team overall strength in the small-ball era.

## Data Availability

The raw data supporting the conclusions of this article will be made available by the authors, without undue reservation.
